# Cultural competence in dementia care: Care workers and managers perspectives on physical and social environments in Long-Term Care

**DOI:** 10.1371/journal.pone.0349763

**Published:** 2026-07-22

**Authors:** Lisa Spang, Katarina Baudin

**Affiliations:** 1 School of Health Sciences, Örebro University, Örebro, Sweden; 2 Division of Occupational Therapy, Department of Neurobiology, Care Sciences and Society, Karolinska Institutet, Huddinge, Sweden; Mae Fah Luang University School of Anti Aging and Regenerative Medicine, THAILAND

## Abstract

This study explores how cultural identity can be preserved in dementia long-term care as resident populations become increasingly diverse. Using a deductive approach informed by the Person–Environment fit framework, the study examines care workers’ and managers’ experiences and strategies across physical and social environments. Findings show that indoor and outdoor settings, communication practices, activities, relationships, and cultural rituals all influence culturally responsive care. Competing priorities such as safety, limited resources, and varying cultural competence, shape the long-term resident’s wellbeing, environment and everyday life. The study highlights the need for interpretation services, structured cultural competence training, and sensory-based approaches to enhance residents’ well-being and inclusion.

## Introduction

Global demographic trends reveal two converging phenomena: accelerated population aging and increasing cultural diversity due to migration. By 2030, one in six people worldwide will be aged 60 years or older, and by 2050, this number will reach 2.1 billion [1]. These shifts are accompanied by longer life expectancy and declining fertility rates, creating unprecedented challenges for health and social care systems globally [2]. Migration further complicates these dynamics, as older adults increasingly live in culturally diverse societies, often with care needs that intersect with linguistic and cultural differences [3].

As individuals age, physical and cognitive decline often necessitates assistance with activities of daily living, including personal hygiene, dressing, household tasks, and healthcare [4]. In Nordic countries, these challenges are amplified by the rising prevalence of dementia among older adults in long-term care. According to The National Board of Health and Welfare in Sweden, approximately 1.2–1.4% of the population were diagnosed with dementia [5], which is around 130,000–150,000 older citizens. Of these, about half (65,000–75,000) lived in long-term care specialized on dementia. However, the forecast for Sweden is that by 2050, the proportion is expected to rise to about 2.2–2.5% of the population [5,6]. Dementia not only affects cognitive functioning but also disrupts emotional well-being and social relationships, making person-centred care essential.

Relocation to long-term care often entails a profound loss of familiar cultural environments, which can negatively impact identity, autonomy, and quality of life [7]. Research consistently demonstrates that meaningful activity and social participation in later life promote health and well-being, reduce depressive symptoms, and may even have stronger effects than pharmacological interventions for certain conditions [8,9]. Moreover, the physical environment plays a crucial role in shaping both the quality of life and the standard of care for older adults living with dementia, while also influencing the caregiving practices of care workers [10–12].

However, despite evidence supporting culturally sensitive and participatory approaches, empirical studies addressing cultural diversity in dementia care remain scarce. A recent systematic review highlights the lack of conceptual frameworks and practical strategies for integrating cultural competence into long-term care [13]. This gap is particularly concerning demographic projections for Europe. The proportion of individuals aged 80 and above in the EU is expected to more than double by 2050, while migration-driven diversity continues to reshape care environments [14]. Residents with dementia from culturally diverse backgrounds often face barriers such as language difficulties, stigma, and limited access to culturally appropriate services [15,16]. These challenges underscore the urgent need for research that explores how dementia care environments can be adapted to respect cultural identity, foster inclusion, and enhance person–environment fit.

The concept of cultural competence, initially articulated by Leininger as the nurse’s ability to understand a patient’s cultural background and values, has since been reconceptualized as an ongoing developmental process [17]. Current scholarship emphasises the interplay between professional knowledge, reflective self-awareness, and intercultural experience in shaping culturally responsive care. The concept has subsequently been further developed and is now understood as an ongoing, evolving process in which professional knowledge, self-awareness, and reflective practice interact with intercultural experience [18].

Emerging frameworks advocate for culturally responsive dementia care through co-design approaches, stakeholder engagement, and adaptation of evidence-based interventions [15,19]. Yet, implementation remains uneven, and addressing these gaps is critical for ensuring equitable, person-centred care in increasingly multicultural long-term care settings.

This study is a part of a larger project and during the initial phase of the project, we identified a significant gap in the theoretical literature regarding how dementia care environments can be adapted to meet multicultural needs. Despite the growing diversity among residents in long-term care facilities, there is a lack of conceptual frameworks and empirical studies that address how dementia care settings can be culturally responsive. This gap is particularly concerning given demographic trends indicating that the number of residents with diverse national origins and culturally specific needs will continue to increase. As such, there is an urgent need for research that explores how dementia care can be designed and delivered in ways that respect and reflect cultural identity, promote inclusion, and enhance person–environment fit. The first step is to explore cultural competence among care workers and managers in dementia care, as they are the ones who can co-create dementia care settings to enhance culturally responsive practices.

Thus, this study aims to *explore care workers´ and managers´ experiences and their strategies for preserving cultural identity when caring for residents from culturally and linguistically diverse backgrounds in dementia long-term care settings*.

## Materials and methods

Ethical approval was granted by the Swedish Ethical Review Authority (Dnr 2024-01131-01). This study employs a deductive analytical approach grounded in the Person–Environment fit (P-E fit) theoretical framework to explore how environmental factors influence the well-being of residents with dementia from culturally diverse background. This approach enables a systematic examination of how specific environmental adaptations may enhance or hinder the person’s sense of comfort, identity, and security. It also facilitates the identification of patterns and gaps in current practices, offering a theoretically informed lens through which culturally responsive dementia care can be evaluated and improved. Further, this study followed the guidelines for qualitative research according to COREQ [20].

### Data collection

This study is part of a research project entitled “Rethinking Environments in Dementia Care Homes (REDem) – Paving the Way for Equality in Care Delivery”, which aims to include immigrants living in Sweden who develop dementia, in culturally adapted Swedish dementia care [21]. In total, 24 individuals were recruited, representing a range of professional roles across ten homes providing long-term care specialized on dementia care, located in four different municipalities in Sweden. Of these facilities, nine were municipally operated and one was privately managed. The size of the facilities varied, with bed capacity ranging from 24 to 174 (mean = 65, median = 52). Participants included eleven unit-managers and 13 care workers. Their educational backgrounds were diverse, encompassing care assistants (with unspecified qualifications), nurse assistants (typically with two years of upper secondary education), as well as professionals with bachelor’s degrees such as registered nurses, occupational therapists, and social welfare officers. Among the care workers, the most common qualification was nurse assistant. Experience in dementia care ranged from 3 to 30 years (mean = 13.7 years), while their length of employment at their current workplace spanned from 2 to 17 years (average = 6.3 years). Data collection was performed though individual semi-structured interviews conducted via digital video conferencing (Microsoft Teams). The interviews were structured around four question areas: the physical environment, social environment, communication, and organisational aspects. Participants were encouraged to reflect on how these question areas were addressed in their everyday work with residents with dementia from culturally diverse backgrounds. All interviews were recorded and lasted between 20 and 33 minutes. Data collection took place between December 2024 and May 2025.

### Ethical statement

Ethical approval was granted by the Swedish Ethical Review Authority (Dnr 2024-01131-01). The participants were informed about the purpose of the study both in writing and orally. All participants signed a consent form before participating in the data collection and were told that participation was voluntary and that they could withdraw their consent at any time during the study, in which case the material from their interview would be removed. They were also informed that the study results would be reported at group level and could not be traced back to any individual.

### Data analysis

To explore how environmental factors align with the needs of residents with dementia from culturally diverse background, we conducted a deductive content analysis based on the methodological steps outlined by Elo and Kyngäs [22]. The theoretical framework guiding the analysis was Person–Environment fit [23,24], which emphasizes the interaction between individual characteristics and environmental conditions. The first step involved constructing a structured matrix with three columns: Person, Environmental Factors, and Fit. This matrix served as the analytical foundation, allowing us to systematically organize and interpret the data. In this study, the “Person” (P) component of the (P–E) fit framework was treated as a static variable. As the analysis focused on a defined group: residents with dementia from culturally diverse background who currently reside in Swedish long-term care. Environment (E) component contained environmental factors, both physical and social. In the second step, “data gathering by content”, all interview content related to environmental factors, both physical and social was extracted and compiled under the “Environmental Factors” column. The third step involved “grouping” the environmental factors into three main domains: physical environment indoors, physical environment outdoors, and social environment. This classification enabled a clearer understanding of how different environmental dimensions contribute to or hinder P-E fit. Finally, in the fourth step, categorisation phase, we analysed the data to identify adaptation strategies that was used to fit between person and environment. This included evaluating various environmental adaptations, such as culturally familiar objects, multilingual communication, or inclusive social activities. This adaption strategies are presented in Table 1 and an example in how data were entered to the analysis matrix in S1 Appendix. An iterative process was used, and the focus switched between the whole and parts of the data during the analysis. The themes that emerged were discussed within the author group until a consensus was reached. To strengthen the trustworthiness of the analysis process, the authors’ preunderstanding was taken into consideration by using critical reflection and slowing down the process of understanding.

**Table 1:** Analyse sheet for P E fit: to find adaption strategies to preserve cultural identity, promote inclusion, and enhance person–environment fit within long term care for residents with dementia from culturally and linguistically diverse backgrounds.

P (Person)	E (Environment)	Fit (Adaptation Strategy)
**Residents with dementia from culturally and linguistically diverse backgrounds**	**Physical Environment – Indoors**	- Decor with culturally familiar items (e.g., textiles, artwork, furniture styles) - Personalised room setup with objects from home country - Use of multilingual signage and labels - Neutral but flexible common areas allowing cultural personalisation
	**Physical Environment – Outdoors**	- Garden areas with plants and scents from home country - Spaces for small group gatherings (e.g., shaded benches, kiosks) - Safe walking paths with sensory cues (colours, textures) - Seasonal decorations reflecting cultural traditions
	**Social Environment – Communication & Interaction**	- Care workers with multilingual skills or cultural competence - Use of music, images, and non-verbal cues for engagement - Access to media in native language (TV, radio, YouTube)
	**Social Environment – Activities & Relationships**	- Theme days/weeks celebrating different cultures (food, music, dance) - Group activities adapted to sensory and cognitive abilities - Inclusion of family and community volunteers from similar backgrounds - Flexible seating arrangements to support social comfort
	**Social Environment – Rituals & Traditions**	- Recognition of cultural holidays and religious practices - Access to culturally appropriate food and drink - Opportunities for spiritual expression (e.g., prayer spaces) - Avoidance of stereotypes; focus on individual preferences

## Results

### Physical environment – indoors

Participants described the process of adapting the indoor physical environment in dementia care homes as complex, primarily due to the need to accommodate a diverse group of residents with varying needs within shared spaces. Several considerations were highlighted, particularly in units designated for individuals with Behavioural and Psychological Symptoms of Dementia (BPSD). In these settings, safety was prioritised, necessitating a minimalist design to avoid environmental triggers and to eliminate objects that could potentially be thrown or damaged.

In standard dementia units, care workers made efforts to create a homely atmosphere. However, participants noted that the aesthetic choices often reflected Swedish cultural preferences, which might not resonate with residents from other cultural backgrounds. When discussing cultural adaptation, participants suggested that small-scale modifications, such as incorporating culturally familiar textiles, artwork, or furniture styles. That could enhance the sense of belonging for residents from diverse backgrounds. A common reflection was:

“*We haven’t really had to think about it much, as we haven’t had many residents from foreign backgrounds. But of course, you could probably set up a little corner somewhere, maybe put out a flag or something*.” *(Manager)*

It was generally perceived as more feasible to personalise individual residents’ own apartments with objects from their country of origin. In contrast, adapting communal areas was seen as more challenging, given the need to ensure that these spaces remained inclusive and appropriate for all residents.

### Physical environment – outdoors

Participants commonly reflected on the potential for culturally adapted outdoor environments by utilising the care home’s garden areas. These spaces were seen as valuable for incorporating plants and scents familiar to residents’ countries of origin, thereby fostering a sense of recognition and comfort. Several participants suggested creating small outdoor settings that residents could associate with their homeland, such as spaces for small group gatherings where individuals from the same cultural background could converse in their native language, enjoy coffee together. Another reflection was to make use of shaded benches and kiosks designed to evoke familiar atmospheres. One participant envisioned a more dynamic use of the garden:

*“We have a large, beautiful garden where we could build a stage and invite cultural associations to come and perform for the residents*.” *(Care worker)*

For facilities with more extensive outdoor areas, participants described the possibility of designing safe walking paths enriched with sensory cues, using colours, textures, and fragrances to stimulate memory and enhance wellbeing. Additionally, the changing seasons were seen as an opportunity to celebrate various cultural festivals outdoors, with efforts made to recreate distinct atmospheres that resonated with residents’ traditions and experiences.

### Social environment – communication & interaction

Participants described the social environment, particularly communication and interaction, as a continuously evolving and dynamic space that required cultural adaptation to meet the individual needs of each resident. The most common strategy for facilitating communication was the deployment of care workers with multilingual skills or cultural competence. These care workers were either assigned to units where residents shared their cultural background or asked to move between units to assist with interpretation. Managers viewed this approach as a recruitment strategy, aiming to employ a diverse workforce capable of supporting residents from various countries. However, care workers often perceived this practice as exploitative, expressing frustration that their primary duties were disrupted by frequent requests to interpret across departments. Furthermore, they described a challenge in matching residents with personnel from the same culture and language. While the interaction between residents and care workers often worked well, family members were dissatisfied when, for example, the care workers belonged to another clan in their country that held lower status or conflicted with their own, or when the care workers was of the wrong gender.

Another method used to support communication was the incorporation of music, visual aids, and non-verbal cues to foster engagement. These tools helped residents associate with familiar topics or contributed to a sense of calm and emotional connection. One participant explained:

*“No one here speaks his language, but it’s not a problem. Cause we usually play media in his native language that he can watch or listen to”. (Care worker)*

These adaptations were seen as essential for enabling meaningful interaction and maintaining emotional wellbeing, especially in linguistically diverse care settings.

### Social environment – activities & relationships

When discussing the social environment in terms of fostering activities and relationships, participants described how cultural adaptation could be achieved through occasional initiatives rather than daily routines. Suggestions included organising themed days or even week-long events celebrating diverse food cultures, music, and dance performances from various countries. However, such activities were generally viewed as logistically challenging to implement on a regular basis. One participant noted:

*“We haven’t really had to use that approach, but I know there are many cultural volunteer organisations or associations that could be invited to help create group activities adapted to sensory and cognitive abilities during themed events.” (Manager)*

The most common form of adaptation, however, involved engaging family members to interpret and facilitate culturally relevant activities for residents from other countries. This approach was seen as both practical and meaningful, allowing for personalised engagement. Another participant reflected:

*“Maybe we need to buy some armchairs so that people from the same country can sit together and talk*.” *(Care worker)*

These reflections highlight the importance of culturally sensitive social programming and the role of both community organisations and family members in supporting meaningful relationships and engagement among residents.

### Social environment – rituals & traditions

Participants discussed potential adaptations to cultural rituals and traditions, emphasising the importance of increased awareness and recognition of cultural holidays and religious practices. However, such adaptations were not commonly implemented in practice. One participant shared:

“*We had a resident who was Muslim, so we arranged a prayer room, but it was never used, so we removed it and haven’t thought about it since. Religion usually isn’t that important to them*.” *(Care worker)*

Religion and religious rituals were generally perceived as less central to residents’ daily lives. Instead, participants more frequently noted a longing for familiar food. Many facilities relied on pre-prepared meals from large-scale kitchens that primarily served traditional Swedish cuisine, which often did not appeal to residents from other cultural backgrounds. As a result, some care workers took personal initiative to prepare meals using spices and ingredients that residents recognised from their home countries. However, such efforts were limited to specific facilities and were not part of a broader organisational strategy, as most facilities were constrained by the standardised offerings of their contracted meal providers. One participant reflected:

*“You shouldn’t assume that someone wants things a certain way just because they’re from another country. Not everyone is that tied to tradition*.” *(Manager)*

Participants emphasised that their person-centred approach focused on understanding residents as they are in the present, rather than relying on assumptions or prioritising what families considered important. They expressed pride in the practice of collecting a resident’s life story during the first month after admission, to build meaningful relationships. However, they also acknowledged that as dementia progresses, much of the individual’s identity may fade, and thus, traditions were often deprioritised in favour of managing symptoms and behaviours, with the primary goal being to maintain a calm and supportive environment.

## Discussion

Our findings highlight the complex interplay between cultural traditions, person-centred care, and organisational constraints within long-term care settings. Below, we elaborate on the key findings of the study and discuss their implications.

### The gap between care workers creativity and organisational capacity

A persistent tension identified in this study concerns the gap between care workers creativity and the organisation’s capacity to implement non-verbal and sensory-based interventions. While frontline practitioners often display strong intuitive and imaginative skills, their ideas are frequently limited by time constraints, staffing pressures and restricted resources. Recent research suggests that digital innovations may help ease these challenges by offering scalable, individualised tools that complement professional expertise. Technologies that support personalised and meaningful activities for people living with dementia, for example, have been shown to reduce care workers workload while fostering engagement and emotional connection [25]. More broadly, developments in artificial intelligence and assistive technologies are beginning to enhance both diagnostic and therapeutic processes in mental health, indicating an expanding role for digital systems in supporting non-verbal and sensory-oriented practices [26]. These trends align with emerging perspectives on AI-enabled compassionate care, which argue that thoughtfully integrated technology can reinforce person-centred approaches rather than replace the relational work of care workers [27]. Incorporating such tools may therefore provide a forward-looking pathway for organisations seeking to translate care workers creativity into sustainable, resource-efficient interventions.

Cultural adaptation of physical environments in dementia Long-Term Care safety, standardisation, and the limits of cultural expression indoors

The findings highlight the complexity of adapting physical environments in dementia long-term care settings to meet diverse cultural and clinical needs. Participants described indoor environmental modifications as particularly challenging in shared spaces, where safety considerations frequently took precedence, especially in units caring for residents with BPSD. Minimalist design strategies aimed at reducing environmental triggers and preventing harm were prioritised, aligning with previous research that emphasizes risk mitigation in dementia care environments [10,12]. However, such safety-driven approaches may unintentionally constrain opportunities for personalization and cultural expression. Our findings further suggest that dementia long-term care settings were largely unprepared to meet residents’ cultural needs, and levels of cultural competence appeared generally low. This indicates a persistent tension between risk management and person-centred principles, where cultural identity risks becoming marginalised within standardised care environments.

### Cultural competence as practice rather than principle

The growing recognition of cultural diversity raises ongoing questions regarding how cultural competence is understood and operationalised in everyday care practices. Several studies stress the importance of cultural competence among care workers in long-term care settings, particularly when supporting older adults with dementia and migration backgrounds [13, 28–30]. Cultural competence has been framed as essential for providing equitable and person-centred care that acknowledges residents’ diverse cultural identities and lived experiences. At the same time, other scholars argue against narrow, checklist-based interpretations of cultural competence, advocating instead for more reflexive approaches rooted in cultural humility, anti-racist practice, awareness of “othering,” and intersectionality in dementia care for older migrants [31–33]. The present findings suggest that cultural competence in the settings studied remained largely aspirational rather than embedded in organisational routines or environmental planning.

### Homeliness, neutrality, and cultural normativity

In standard dementia units, care workers described efforts to create homelike atmospheres; however, these aesthetic choices predominantly reflected Swedish cultural norms. While participants mentioned small-scale adaptations such as incorporating culturally familiar textiles, flagpoles, or artwork these were perceived as optional rather than integral components of care. Concerns about maintaining neutrality in shared environments often limit such initiatives. This raises critical questions regarding inclusivity, as cultural familiarity has been shown to foster emotional security, belonging, and identity continuity among residents with diverse backgrounds [34,35]. The prioritisation of cultural neutrality may therefore inadvertently reproduce cultural normativity, privileging majority cultural expressions while rendering minority identities less visible.

### Outdoor environments as flexible spaces for cultural engagement

Compared to indoor settings, outdoor environments emerged as a more promising arena for cultural adaptation. Gardens were described as flexible spaces that enabled sensory engagement and social interaction, offering opportunities to incorporate plants, scents, and design elements associated with residents’ cultural heritage. These findings align with existing evidence linking nature-based activities to improved well-being and reduced agitation in dementia care [36–39]. Participants also envisioned outdoor areas as dynamic cultural spaces for seasonal celebrations, music, and performances, potentially strengthening community ties and supporting identity continuity. However, these ideas were largely aspirational, revealing a gap between care workers’ creativity and organisational capacity to implement culturally responsive environmental strategies.

### Social environments and everyday cultural negotiations linguistic diversity and the burden of informal interpretation

Communication and interaction were described as dynamic and evolving processes shaped by linguistic and cultural diversity. Participants highlighted the frequent reliance on multilingual care workers to support communication, either through targeted recruitment or informal cross-unit assistance. While managers viewed this as a pragmatic approach to inclusivity, care workers often experienced these expectations as burdensome, noting how frequent interpretation requests disrupted their core caregiving responsibilities.

This tension reflects broader concerns in the literature regarding the sustainability of informal interpretation practices in healthcare and their impact on workload and job satisfaction [40,41]. The findings underscore the need for organisational policies that introduce formal interpretation services and promote the use of non-verbal and sensory-based communication strategies, rather than excessive reliance on care workers with language competence alone.

### The limits of cultural matching

Matching residents with care workers from similar cultural or linguistic backgrounds was described as desirable but rarely feasible. Participants also reported that cultural similarity did not necessarily guarantee positive interactions, as family members sometimes expressed dissatisfaction related to social hierarchies, gender norms, or historical conflicts within residents’ countries of origin. These findings underscore the complexity and internal diversity of cultural identities and suggest that simplistic matching strategies may overlook deeper relational dynamics, an issue previously identified in research on intercultural care relationships [42].

### Beyond language: Sensory and non-verbal pathways to connection

Care workers described using music, visual aids, and non-verbal cues to foster emotional connection and reduce anxiety, particularly for residents with limited verbal abilities. Such strategies were perceived as effective and align with evidence supporting the therapeutic role of music and sensory stimulation in dementia care [43,44]. Interestingly, participants with lower levels of cultural competence sometimes downplayed the importance of language, emphasising instead that declining communication abilities were common across all residents. While this perspective reflects clinical realities of dementia progression, it may also risk obscuring the cultural significance of language and communication preferences. This highlights a conceptual tension in how the “Person” component of P–E fit is understood: while cultural identity is often treated as a stable attribute [23], the progression of dementia means that aspects of the individual’s identity, including culturally shaped communication patterns, may gradually diminish. Acknowledging this dynamic process adds important nuance to the interpretation of care workers´ experiences and underscores the need for flexible, evolving approaches to cultural responsiveness in advanced dementia care.

Cultural meaning in daily life: rituals, food, and identity cultural awareness without structural integration

Participants acknowledged the importance of cultural awareness related to holidays, religious practices, and food preferences; however, these considerations were rarely integrated into everyday routines. This discrepancy suggests that cultural sensitivity functions more as an ideal than an operational practice within many facilities, echoing previous research emphasizing cultural competence as critical, yet inconsistently implemented, in dementia care [42].

### Religious and spiritual practices: Visible absence, latent significance

Religious rituals were generally perceived as peripheral to daily care, reflecting assumptions that spirituality diminishes with advanced age or cognitive impairment [45]. The unused prayer room exemplifies the tension between proactive accommodation and assumptions about residents’ priorities. While some residents may no longer actively request spiritual support, others may still derive comfort from rituals and symbols, a complexity noted in literature on barriers to spiritual care in long-term care settings [46–48]. Integrating spiritual care into dementia long-term care has been associated with positive outcomes for both residents and care environments [45], suggesting that its marginalization reflects broader gaps in care quality and consistency in Sweden.

### Food as a central marker of cultural identity

Food emerged as a more salient cultural marker than religion, reinforcing previous research highlighting diet as central to identity, well-being, and quality of life in later life [49,50]. Beyond nutrition, food practices carry symbolic, aesthetic, and social meanings tied to culture and belonging [51–53]. For many long-term care residents, meals constitute the highlight of the day and provide vital opportunities for social interaction and relationship-building with caregivers [54]. Participants described ad hoc efforts to prepare culturally familiar meals; however, these initiatives depended heavily on individual commitment rather than organizational support. Reliance on standardized meal providers limited flexibility, reflecting barriers commonly identified in studies of organizational culture change in dementia care [55–57].

### Person-centred care and the risk of cultural discontinuity

Participants emphasised a person-centred approach prioritizing residents’ present identity rather than past traditions or family expectations. While consistent with best-practice dementia care [58], this orientation risks deprioritising cultural continuity as cognitive decline progresses. These findings point to the need for nuanced strategies that balance symptom management with ongoing opportunities for cultural expression, even in advanced stages of dementia. Future research should explore how design guidelines and policy frameworks can better integrate cultural competence into environmental planning. This may involve participatory approaches that engage residents, families, and cultural associations in co-design processes, ensuring that physical spaces support both clinical needs and cultural identity. Future research should also explore how systemic changes, such as flexible meal contracts or structured cultural competence training among the care workers which might enable more consistent integration of cultural practices into person-centred care [59].

### Methodological considerations

The methodological choices in this study warrant careful consideration in relation to both their strengths and limitations. The deductive analytical approach, grounded in the Person–Environment fit framework, provided a robust theoretical lens through which the interaction between individual characteristics and environmental conditions could be systematically examined. By treating the “Person” component as a static variable, the analysis was able to focus on a clearly defined group, residents with dementia from culturally diverse background, thus ensuring conceptual clarity. However, this decision also constrained the exploration of individual variability within the group, potentially overlooking nuances in personal agency and adaptation. The sample was diverse in terms of professional roles and educational backgrounds, which strengthened the breadth of perspectives captured. Nevertheless, the imbalance between unit managers and care workers raises questions about representativeness. Managers may have provided more organisationally oriented accounts, while the voices of care assistants and nurse assistants, who often have the most direct contact with residents were comparatively underrepresented. This imbalance could have influenced the findings regarding structural and managerial perspectives rather than everyday relational practices.

The deductive content analysis, guided by Elo and Kyngäs [22], ensured systematic organisation of data and facilitated the identification of adaptation strategies relevant to P–E fit. The structured matrix was particularly useful in maintaining analytical coherence. While the iterative process and critical reflection within the author group enhanced trustworthiness, the researchers’ professional backgrounds as occupational therapists may have introduced interpretive biases, particularly in privileging environmental and functional aspects of care. The explicit acknowledgement of preunderstanding and the deliberate slowing down of the interpretive process were important steps in mitigating this risk.

Finally, adherence to COREQ guidelines strengthened the transparency and rigour of the qualitative design. Yet, as with all qualitative research, the findings are contextually bound to Swedish dementia care homes and may not be directly transferable to other cultural or organisational settings. The emphasis on culturally diverse residents is a notable strength, but further research involving residents themselves, rather than solely care workers perspectives, would provide a more comprehensive understanding of person–environment fit in dementia care.

## Conclusion

Overall, the results suggest that P-E fit in dementia long-term care are affected and shaped by competing priorities, such as safety, resource limitations, and variant cultural competence such as embedded standardised meal contracts and facility-wide protocols to reduce reliance on individual care workers commitment and ensure more consistent, equitable practices.

Also, the results suggest that culturally responsive communication in dementia long-term care requires a multifaceted approach that goes beyond language matching. Organisational policies should include dedicated cultural mediator roles or protected time for interpretation to avoid the exploitative reliance on multilingual care workers, alongside structured cultural competence training and the integration of non-verbal and sensory-based interventions. Future research should also explore how these strategies can be systematically implemented without overburdening care workers, and how they influence residents’ quality of life and emotional well-being.

## Supporting information

S1 Appendix An example of how data were entered into the analysis matrix. (DOCX)
