# Supplementary material for: Cultural competence in dementia care: Care workers and managers perspectives on physical and social environments in Long-Term Care
Source: PLoS One. 2026 Jul 22;21(7):e0349763. doi: 10.1371/journal.pone.0349763 (PMC13390856; doi:10.1371/journal.pone.0349763)
Supplement: S1 Appendix — (DOCX) [file pone.0349763.s001.docx]

| **Appendix 1, an example of how data were entered into the analysis matrix** | | |
| --- | --- | --- |
| **P (Person)** | **E (Environment)** | **Fit (Adaptation Strategy)** |
| Residents with dementia from culturally and linguistically diverse backgrounds | Physical Environment – Indoors  “No, it is not really possible to do that (to adapt it culturally). The corridors are narrow and very long, and there is no space to make any changes in them. Not even in one of the units, because there the apartments are located directly along the corridor. So in that sense, it is more up to the resident to furnish their own apartment in a style that suits them, so to speak.” (data unit 1 from manager)  “I mean, I really believe in using pictures or objects that feel like home. It could be something like a particular candle holder—of course we won’t light real candles—but perhaps a lantern or whatever it may be that is very specific to that individual person.” (Data unit 2 from staff) | - Decor with culturally familiar items (e.g., textiles, artwork, furniture styles) - Personalized room setup with objects from home country - Use of multilingual signage and labels - Neutral but flexible common areas allowing cultural personalization |
