# Supplementary material for: Cultural competence in dementia care: Care workers and managers perspectives on physical and social environments in Long-Term Care
Source: PLoS One. 2026 Jul 22;21(7):e0349763. doi: 10.1371/journal.pone.0349763 (PMC13390856; doi:10.1371/journal.pone.0349763)
Supplement: Peer review history

# Peer review: Decision Letter 0

**DOI:** 10.1371/journal.pone.0349763.r001

**Authors:** Assoc. Prof. Phakkharawat Sittiprapaporn

19 Mar 2026

PONE-D-26-03403

Cultural competence in dementia care: Care workers and managers perspectives on physical and social environments in Long-Term Care

PLOS One

Dear Dr. Spang,

Thank you for submitting your manuscript to PLOS ONE. After careful consideration, we feel that it has merit but does not fully meet PLOS ONE’s publication criteria as it currently stands. Therefore, we invite you to submit a revised version of the manuscript that addresses the points raised during the review process.

Please submit your revised manuscript by May 03 2026 11:59PM. If you will need more time than this to complete your revisions, please reply to this message or contact the journal office at plosone@plos.org. When you're ready to submit your revision, log on to https://www.editorialmanager.com/pone/ and select the 'Submissions Needing Revision' folder to locate your manuscript file.

Please include the following items when submitting your revised manuscript:

- A letter that responds to each point raised by the academic editor and reviewer(s). You should upload this letter as a separate file labeled 'Response to Reviewers'.
- A marked-up copy of your manuscript that highlights changes made to the original version. You should upload this as a separate file labeled 'Revised Manuscript with Track Changes'.
- An unmarked version of your revised paper without tracked changes. You should upload this as a separate file labeled 'Manuscript'.

If you would like to make changes to your financial disclosure, please include your updated statement in your cover letter. Guidelines for resubmitting your figure files are available below the reviewer comments at the end of this letter.

If applicable, we recommend that you deposit your laboratory protocols in protocols.io to enhance the reproducibility of your results. Protocols.io assigns your protocol its own identifier (DOI) so that it can be cited independently in the future. For instructions see: https://journals.plos.org/plosone/s/submission-guidelines#loc-laboratory-protocols. Additionally, PLOS ONE offers an option for publishing peer-reviewed Lab Protocol articles, which describe protocols hosted on protocols.io. Read more information on sharing protocols at https://plos.org/protocols?utm_medium=editorial-email&utm_source=authorletters&utm_campaign=protocols.

We look forward to receiving your revised manuscript.

Kind regards,

Assoc. Prof. Phakkharawat Sittiprapaporn, Ph.D.

Academic Editor

PLOS One

Journal Requirements:

When submitting your revision, we need you to address these additional requirements.

1. Please ensure that your manuscript meets PLOS ONE's style requirements, including those for file naming. The PLOS ONE style templates can be found at

https://journals.plos.org/plosone/s/file?id=wjVg/PLOSOne_formatting_sample_main_body.pdf and https://journals.plos.org/plosone/s/file?id=ba62/PLOSOne_formatting_sample_title_authors_affiliations.pdf

2. In your ethics statement, please ensure you have provided the full name of the ethics committee(s) that approved this study. If more than 5 committees provided approval, it is acceptable to provide a list as a Supporting Information file.

3. Please provide additional details regarding participant consent. In the ethics statement in the Methods and online submission information, please ensure that you have specified (1) whether consent was informed and (2) what type you obtained (for instance, written or verbal, and if verbal, how it was documented and witnessed). If your study included minors, state whether you obtained consent from parents or guardians. If the need for consent was waived by the ethics committee, please include this information.

If you are reporting a retrospective study of medical records or archived samples, please ensure that you have discussed whether all data were fully anonymized before you accessed them and/or whether the IRB or ethics committee waived the requirement for informed consent. If patients provided informed written consent to have data from their medical records used in research, please include this information.

4. Thank you for stating in your Funding Statement:

[This research was supported by “Forte” (Swedish Research Council for Health, Working Life and Welfare), under the Swedish Ministry of Health and Social Affairs. Dnr: (2023-00171.).].

Please provide an amended statement that declares *all* the funding or sources of support (whether external or internal to your organization) received during this study, as detailed online in our guide for authors at http://journals.plos.org/plosone/s/submit-now.  Please also include the statement “There was no additional external funding received for this study.” in your updated Funding Statement.

Please include your amended Funding Statement within your cover letter. We will change the online submission form on your behalf.

5. Please note that funding information should not appear in any section or other areas of your manuscript. We will only publish funding information present in the Funding Statement section of the online submission form. Please remove any funding-related text from the manuscript.

6. We note that you have indicated that there are restrictions to data sharing for this study. For studies involving human research participant data or other sensitive data, we encourage authors to share de-identified or anonymized data. However, when data cannot be publicly shared for ethical reasons, we allow authors to make their data sets available upon request. For information on unacceptable data access restrictions, please see http://journals.plos.org/plosone/s/data-availability#loc-unacceptable-data-access-restrictions.

Before we proceed with your manuscript, please address the following prompts:

a) If there are ethical or legal restrictions on sharing a de-identified data set, please explain them in detail (e.g., data contain potentially identifying or sensitive patient information, data are owned by a third-party organization, etc.) and who has imposed them (e.g., a Research Ethics Committee or Institutional Review Board, etc.). Please also provide contact information for a data access committee, ethics committee, or other institutional body to which data requests may be sent.

b) If there are no restrictions, please upload the minimal anonymized data set necessary to replicate your study findings to a stable, public repository and provide us with the relevant URLs, DOIs, or accession numbers. Please see http://www.bmj.com/content/340/bmj.c181.long for guidelines on how to de-identify and prepare clinical data for publication. For a list of recommended repositories, please see https://journals.plos.org/plosone/s/recommended-repositories. You also have the option of uploading the data as Supporting Information files, but we would recommend depositing data directly to a data repository if possible.

Please update your Data Availability statement in the submission form accordingly.

7. Please include your full ethics statement in the ‘Methods’ section of your manuscript file. In your statement, please include the full name of the IRB or ethics committee who approved or waived your study, as well as whether or not you obtained informed written or verbal consent. If consent was waived for your study, please include this information in your statement as well.

8. We note that this manuscript consists of interview transcripts. Can you please confirm that all participants gave consent for interview transcript to be published?

If they DID provide consent for these transcripts to be published, please also confirm that the transcripts do not contain any potentially identifying information (or let us know if the participants consented to having their personal details published and made publicly available). We consider the following details to be identifying information:

- Names, nicknames, and initials

- Age more specific than round numbers

- GPS coordinates, physical addresses, IP addresses, email addresses

- Information in small sample sizes (e.g. 40 students from X class in X year at X university)

- Specific dates (e.g. visit dates, interview dates)

- ID numbers

Or, if the participants DID NOT provide consent for these transcripts to be published:

- Provide a de-identified version of the data or excerpts of interview responses

- Provide information regarding how these transcripts can be accessed by researchers who meet the criteria for access to confidential data, including:

a) the grounds for restriction

b) the name of the ethics committee, Institutional Review Board, or third-party organization that is imposing sharing restrictions on the data

c) a non-author, institutional point of contact that is able to field data access queries, in the interest of maintaining long-term data accessibility.

d) Any relevant data set names, URLs, DOIs, etc. that an independent researcher would need in order to request your minimal data set.

For further information on sharing data that contains sensitive participant information, please see: https://journals.plos.org/plosone/s/data-availability#loc-human-research-participant-data-and-other-sensitive-data

If there are ethical, legal, or third-party restrictions upon your dataset, you must provide all of the following details (https://journals.plos.org/plosone/s/data-availability#loc-acceptable-data-access-restrictions):

1. A complete description of the dataset

2. The nature of the restrictions upon the data (ethical, legal, or owned by a third party) and the reasoning behind them

3. The full name of the body imposing the restrictions upon your dataset (ethics committee, institution, data access committee, etc.)

4. If the data are owned by a third party, confirmation of whether the authors received any special privileges in accessing the data that other researchers would not have

5. Direct, non-author contact information (preferably email) for the body imposing the restrictions upon the data, to which data access requests can be sent

If the reviewer comments include a recommendation to cite specific previously published works, please review and evaluate these publications to determine whether they are relevant and should be cited. There is no requirement to cite these works unless the editor has indicated otherwise.

[Note: HTML markup is below. Please do not edit.]

Reviewers' comments:

Reviewer's Responses to Questions

**Comments to the Author**

1. Is the manuscript technically sound, and do the data support the conclusions?

The manuscript must describe a technically sound piece of scientific research with data that supports the conclusions. Experiments must have been conducted rigorously, with appropriate controls, replication, and sample sizes. The conclusions must be drawn appropriately based on the data presented.

Reviewer #1: Yes

Reviewer #2: Yes

**********

2. Has the statistical analysis been performed appropriately and rigorously?

Reviewer #1: Yes

Reviewer #2: Yes

**********

3. Have the authors made all data underlying the findings in their manuscript fully available?

The PLOS Data policy requires authors to make all data underlying the findings described in their manuscript fully available without restriction, with rare exception (please refer to the Data Availability Statement in the manuscript PDF file). The data should be provided as part of the manuscript or its supporting information, or deposited to a public repository. For example, in addition to summary statistics, the data points behind means, medians and variance measures should be available. If there are restrictions on publicly sharing data—e.g. participant privacy or use of data from a third party—those must be specified.

Reviewer #1: Yes

Reviewer #2: Yes

**********

4. Is the manuscript presented in an intelligible fashion and written in standard English?

PLOS ONE does not copyedit accepted manuscripts, so the language in submitted articles must be clear, correct, and unambiguous. Any typographical or grammatical errors should be corrected at revision, so please note any specific errors here.

Reviewer #1: Yes

Reviewer #2: Yes

**********

5. Review Comments to the Author

Reviewer #1: Hello. Thank you for the opportunity to review this article- as it is a very important and meaningful topic to research. Here are some thoughts/questions I had:

1) I feel it would help to have more context and detail about the people who were interviewed. You mention difference in opinions based on their jobs (managers vs care workers) but I didn't see that in the results. There are a few quotes but could do better with more information about the not just who were making the comments but the variety of comments that you received. It could really help to differentiate between your results and discussion as there appeared to be quite a bit of repetition in both. The discussion section can be made less repetitive

2) How do you define cultural competence? Perhaps more discussion of it could be helpful for the reader to understand some of the terms you have used.

3) It would be helpful to note if the people you interviewed were from diverse ethnic backgrounds or were immigrants themselves, etc.

4) Could you explain a bit about how the facilities/ people were chosen/ selected? Was there a difference in the opinions based on municipal vs privately managed?

5) I see an "insert table 1 here" message but I don't see the table on page 7

6) You mention voices of care assistances were underrepresented- can you explain that?

7) There are many grammatical/ language. Please do spell check and ensure singular/ plural is corrected. For example: Residents from diverse background(s), adaptation strategies vs adaption strategies, specialized "in" dementia care, etc. There some instances of missing punctuations as well.

Reviewer #2: This manuscript addresses a timely and critical gap in geriatric care by exploring how long-term care (LTC) environments can be adapted to preserve the cultural identity of diverse resident populations living with dementia. Utilizing the Person–Environment (P-E) fit framework, the authors provide a structured exploration of how physical and social factors influence culturally responsive care within the Swedish context. I recommend a Minor Revision to address specific areas regarding participant representation, the presentation of findings, and the depth of the discussion.

1- In the methodological considerations, you rightly acknowledge the imbalance between unit managers (n=11) and care workers (n=13). To strengthen the manuscript, please add a brief sentence in the Discussion section exploring how this imbalance might have shaped the perspective of the findings on "organizational capacity" versus "everyday practices.

2- Table 1 serves as an excellent analytical foundation. However, it currently reads more like a matrix of potential strategies. I recommend adding a few specific illustrative examples from the interviews (e.g., the specific use of "kiosks" or "shaded benches" mentioned in your results) directly into the "Fit" column to make it a more practical tool for readers.

3- The author mentions that for the analysis, the "Person" component of the P-E fit was treated as a static variable. While this provides conceptual clarity, your results highlight that "as dementia progresses, much of the individual’s identity may fade". Briefly discussing the tension between treating the person as a static cultural entity versus the dynamic nature of cognitive decline would add significant theoretical depth to the Discussion.

4- Manuscript findings regarding the "exploitative" feel of informal interpretation among multilingual staff are a major highlight. In the Conclusion, please be more explicit in recommending specific organizational policies (e.g., dedicated "cultural mediator" roles or protected time for interpretation) to prevent staff burnout, rather than just calling for "formal interpretation services" generally.

5- The results indicate that many adaptations (like preparing culturally familiar food) are dependent on individual initiative rather than systemic support. I suggest adding a sentence in the Conclusion emphasizing that cultural competence should be integrated into standardized meal contracts and facility-wide protocols to move beyond reliance on individual staff commitment.

6- To enhance the Discussion regarding the gap between staff creativity and organizational capacity, the authors could benefit from exploring how digital innovations might bridge these resource limitations. The following references are suggested to be introduced in this section to provide a forward-looking technological framework for the study’s conclusions on non-verbal and sensory-based interventions:

• Goodall, G., Taraldsen, K., & Serrano, J. A. (2021). The use of technology in creating individualized, meaningful activities for people living with dementia: A systematic review. Dementia, 20(4), 1442-1469.

• Abrar, M., Khalid, S., & Ullah, R. (2026). The intersection of artificial intelligence and assistive technologies in the diagnosis and intervention of mental health conditions. Artificial Intelligence Review, 59(2), 40.

• Dawod, O. Y. (2025). Exploring the impact of artificial intelligence on person-centered compassionate care: Implications for patients, families, and caregivers. Transnational Medical and Health Sciences Journal, 1(1), 12-20.

**********

6. PLOS authors have the option to publish the peer review history of their article (what does this mean?). If published, this will include your full peer review and any attached files.

If you choose “no”, your identity will remain anonymous but your review may still be made public.

**Do you want your identity to be public for this peer review?**  For information about this choice, including consent withdrawal, please see our Privacy Policy.

Reviewer #1: No

Reviewer #2: No

**********

[NOTE: If reviewer comments were submitted as an attachment file, they will be attached to this email and accessible via the submission site. Please log into your account, locate the manuscript record, and check for the action link "View Attachments". If this link does not appear, there are no attachment files.]

To ensure your figures meet our technical requirements, please review our figure guidelines: https://journals.plos.org/plosone/s/figures

You may also use PLOS’s free figure tool, NAAS, to help you prepare publication quality figures: https://journals.plos.org/plosone/s/figures#loc-tools-for-figure-preparation.

NAAS will assess whether your figures meet our technical requirements by comparing each figure against our figure specifications.

# Author comment: Author response to Decision Letter 1

**DOI:** 10.1371/journal.pone.0349763.r002

24 Apr 2026

Reviewer #1: Hello. Thank you for the opportunity to review this article- as it is a very important and meaningful topic to research. Here are some thoughts/questions I had:

1) I feel it would help to have more context and detail about the people who were interviewed. You mention difference in opinions based on their jobs (managers vs care workers) but I didn't see that in the results. There are a few quotes but could do better with more information about the not just who were making the comments but the variety of comments that you received. It could really help to differentiate between your results and discussion as there appeared to be quite a bit of repetition in both. The discussion section can be made less repetitive

Answer: Thank you for this insight. The discussion has been reviewed and adjusted to remove repetitions, and the quotations have been clarified by specifying the professional role of the participants.

2) How do you define cultural competence? Perhaps more discussion of it could be helpful for the reader to understand some of the terms you have used.

Answer: Thank you for your comment. We have chosen to introduce the term cultural competence in the introduction, but in the discussion the concept is defined in relation to the study’s findings, as it emerged as one of the central themes in the results.

3) It would be helpful to note if the people you interviewed were from diverse ethnic backgrounds or were immigrants themselves, etc.

Answer: Thank you for the comment. It would indeed have been interesting, but this is not information we have in the present study. However, we will consider it in future research

4) Could you explain a bit about how the facilities/ people were chosen/ selected? Was there a difference in the opinions based on municipal vs privately managed?

Answer: Thank you for bringing this to our attention. The participating care homes were invited as part of the larger project, which is already described in the data collection section. Regarding how the facilities differed from one another, we could not identify any clear patterns; the perspectives expressed appeared to relate more to individual experiences and professional backgrounds than to geographical factors or differences between municipal and privately operated settings. This point may therefore be best addressed directly in the response to the reviewer rather than incorporated into the manuscript. This is stated in the section of Data collection

5) I see an "insert table 1 here" message but I don't see the table on page 7

Answer: Yes, this was an instruction intended for the editor

6) You mention voices of care assistances were underrepresented- can you explain that?

Answer: Thank you for bringing this to our attention. In Swedish dementia care, typically care workers consist of both assistant nurses and care assistants, but assistant nurses make up the majority of the workforce. In our sample, this distribution was reflected in the recruitment: most participating staff members were assistant nurses or registered nurses, while only a small number were care assistants. This explains why the voices of care assistants were underrepresented in the study.

7) There are many grammatical/ language. Please do spell check and ensure singular/ plural is corrected. For example: Residents from diverse background(s), adaptation strategies vs adaption strategies, specialized "in" dementia care, etc. There some instances of missing punctuations as well.

Answer: Thank you for pointing this out. We have reviewed the manuscript once more with a focus on language and have made minor corrections.

Reviewer #2: This manuscript addresses a timely and critical gap in geriatric care by exploring how long-term care (LTC) environments can be adapted to preserve the cultural identity of diverse resident populations living with dementia. Utilizing the Person–Environment (P-E) fit framework, the authors provide a structured exploration of how physical and social factors influence culturally responsive care within the Swedish context. I recommend a Minor Revision to address specific areas regarding participant representation, the presentation of findings, and the depth of the discussion.

1- In the methodological considerations, you rightly acknowledge the imbalance between unit managers (n=11) and care workers (n=13). To strengthen the manuscript, please add a brief sentence in the Discussion section exploring how this imbalance might have shaped the perspective of the findings on "organizational capacity" versus "everyday practices.

Answer: Thank you for bringing this to our attention, this is an exciting perspective that emerged in the results, and we are preparing a separate manuscript focusing specifically on this topic. In the present manuscript, we have chosen to address it only in methodological discussion.

2- Table 1 serves as an excellent analytical foundation. However, it currently reads more like a matrix of potential strategies. I recommend adding a few specific illustrative examples from the interviews (e.g., the specific use of "kiosks" or "shaded benches" mentioned in your results) directly into the "Fit" column to make it a more practical tool for readers.

Answer: Thank you for this valuable insight. To illustrate how the matrix was used, an appendix has been added in the end of the manuscript, the Revised Manuscript with Tracked Changes.

3- The author mentions that for the analysis, the "Person" component of the P-E fit was treated as a static variable. While this provides conceptual clarity, your results highlight that "as dementia progresses, much of the individual’s identity may fade". Briefly discussing the tension between treating the person as a static cultural entity versus the dynamic nature of cognitive decline would add significant theoretical depth to the Discussion.

Answer: We agree that this is important to address in the discussion, and an addition has been made to the final paragraph under the subheading Communication and interaction.

4- Manuscript findings regarding the "exploitative" feel of informal interpretation among multilingual staff are a major highlight. In the Conclusion, please be more explicit in recommending specific organizational policies (e.g., dedicated "cultural mediator" roles or protected time for interpretation) to prevent staff burnout, rather than just calling for "formal interpretation services" generally.

Answer: Thank you, the conclusion has been improved according to your suggestion.

5- The results indicate that many adaptations (like preparing culturally familiar food) are dependent on individual initiative rather than systemic support. I suggest adding a sentence in the Conclusion emphasizing that cultural competence should be integrated into standardized meal contracts and facility-wide protocols to move beyond reliance on individual staff commitment.

Answer: We agree with this suggested improvement, and this has been clarified in the conclusion.

6- To enhance the Discussion regarding the gap between staff creativity and organizational capacity, the authors could benefit from exploring how digital innovations might bridge these resource limitations. The following references are suggested to be introduced in this section to provide a forward-looking technological framework for the study’s conclusions on non-verbal and sensory-based interventions:

• Goodall, G., Taraldsen, K., & Serrano, J. A. (2021). The use of technology in creating individualized, meaningful activities for people living with dementia: A systematic review. Dementia, 20(4), 1442-1469.

• Abrar, M., Khalid, S., & Ullah, R. (2026). The intersection of artificial intelligence and assistive technologies in the diagnosis and intervention of mental health conditions. Artificial Intelligence Review, 59(2), 40.

• Dawod, O. Y. (2025). Exploring the impact of artificial intelligence on person-centered compassionate care: Implications for patients, families, and caregivers. Transnational Medical and Health Sciences Journal, 1(1), 12-20.

Answer: Thank you for this insight and perspective. The suggested references have been included and a discussion has been added at the beginning of the discussion section, building up to the conclusion on non-verbal and sensory-based interventions.

Attachment Submitted filename: Response to Reviewers.docx

# Peer review: Decision Letter 1

**DOI:** 10.1371/journal.pone.0349763.r003

**Authors:** Assoc. Prof. Phakkharawat Sittiprapaporn

5 May 2026

Cultural competence in dementia care: Care workers and managers perspectives on physical and social environments in Long-Term Care

PONE-D-26-03403R1

Dear Dr. Spang,

We’re pleased to inform you that your manuscript has been judged scientifically suitable for publication and will be formally accepted for publication once it meets all outstanding technical requirements.

Within one week, you’ll receive an e-mail detailing the required amendments. When these have been addressed, you’ll receive a formal acceptance letter and your manuscript will be scheduled for publication.

An invoice will be generated when your article is formally accepted. Please note, if your institution has a publishing partnership with PLOS and your article meets the relevant criteria, all or part of your publication costs will be covered. Please make sure your user information is up-to-date by logging into Editorial Manager at Editorial Manager® and clicking the ‘Update My Information' link at the top of the page. For questions related to billing, please contact billing support.

If your institution or institutions have a press office, please notify them about your upcoming paper to help maximize its impact. If they’ll be preparing press materials, please inform our press team as soon as possible -- no later than 48 hours after receiving the formal acceptance. Your manuscript will remain under strict press embargo until 2 pm Eastern Time on the date of publication. For more information, please contact onepress@plos.org.

Kind regards,

Assoc. Prof. Phakkharawat Sittiprapaporn, Ph.D.

Academic Editor

PLOS One

Additional Editor Comments (optional):

Reviewers' comments:

Reviewer's Responses to Questions

**Comments to the Author**

1. If the authors have adequately addressed your comments raised in a previous round of review and you feel that this manuscript is now acceptable for publication, you may indicate that here to bypass the “Comments to the Author” section, enter your conflict of interest statement in the “Confidential to Editor” section, and submit your "Accept" recommendation.

Reviewer #2: All comments have been addressed

**********

2. Is the manuscript technically sound, and do the data support the conclusions?

The manuscript must describe a technically sound piece of scientific research with data that supports the conclusions. Experiments must have been conducted rigorously, with appropriate controls, replication, and sample sizes. The conclusions must be drawn appropriately based on the data presented.

Reviewer #2: Yes

**********

3. Has the statistical analysis been performed appropriately and rigorously?

Reviewer #2: Yes

**********

4. Have the authors made all data underlying the findings in their manuscript fully available?

The PLOS Data policy requires authors to make all data underlying the findings described in their manuscript fully available without restriction, with rare exception (please refer to the Data Availability Statement in the manuscript PDF file). The data should be provided as part of the manuscript or its supporting information, or deposited to a public repository. For example, in addition to summary statistics, the data points behind means, medians and variance measures should be available. If there are restrictions on publicly sharing data—e.g. participant privacy or use of data from a third party—those must be specified.

Reviewer #2: Yes

**********

5. Is the manuscript presented in an intelligible fashion and written in standard English?

PLOS ONE does not copyedit accepted manuscripts, so the language in submitted articles must be clear, correct, and unambiguous. Any typographical or grammatical errors should be corrected at revision, so please note any specific errors here.

Reviewer #2: Yes

**********

6. Review Comments to the Author

Please use the space provided to explain your answers to the questions above. You may also include additional comments for the author, including concerns about dual publication, research ethics, or publication ethics. (Please upload your review as an attachment if it exceeds 20,000 characters)

Reviewer #2: (No Response)

**********

7. PLOS authors have the option to publish the peer review history of their article (what does this mean?). If published, this will include your full peer review and any attached files.

If you choose “no”, your identity will remain anonymous but your review may still be made public.

**Do you want your identity to be public for this peer review?**  For information about this choice, including consent withdrawal, please see our Privacy Policy.

Reviewer #2: No

**********

# Editor report: Acceptance letter

**DOI:** 10.1371/journal.pone.0349763.r004

**Authors:** Assoc. Prof. Phakkharawat Sittiprapaporn

PONE-D-26-03403R1

PLOS One

Dear Dr. Spang,

I'm pleased to inform you that your manuscript has been deemed suitable for publication in PLOS One. Congratulations! Your manuscript is now being handed over to our production team.

At this stage, our production department will prepare your paper for publication. This includes ensuring the following:

* All references, tables, and figures are properly cited

* All relevant supporting information is included in the manuscript submission,

* There are no issues that prevent the paper from being properly typeset

You will receive further instructions from the production team, including instructions on how to review your proof when it is ready. Please keep in mind that we are working through a large volume of accepted articles, so please give us a few days to review your paper and let you know the next and final steps.

Lastly, if your institution or institutions have a press office, please let them know about your upcoming paper now to help maximize its impact. If they'll be preparing press materials, please inform our press team within the next 48 hours. Your manuscript will remain under strict press embargo until 2 pm Eastern Time on the date of publication. For more information, please contact onepress@plos.org.

You will receive an invoice from PLOS for your publication fee after your manuscript has reached the completed accept phase. If you receive an email requesting payment before acceptance or for any other service, this may be a phishing scheme. Learn how to identify phishing emails and protect your accounts at https://explore.plos.org/phishing.

If we can help with anything else, please email us at customercare@plos.org.

Thank you for submitting your work to PLOS ONE and supporting open access.

Kind regards,

PLOS ONE Editorial Office Staff

on behalf of

Assoc. Prof. Dr. Assoc. Prof. Phakkharawat Sittiprapaporn

Academic Editor

PLOS One
